# Outcomes of patients aged ≥80 years with respiratory failure initially treated with non-invasive ventilation in European intensive care units before and during COVID-19 pandemic

**DOI:** 10.1186/s13613-023-01173-2

**Published:** 2023-09-12

**Authors:** Kamil Polok, Jakub Fronczek, Bertrand Guidet, Antonio Artigas, Dylan W. De Lange, Jesper Fjølner, Susannah Leaver, Michael Beil, Sigal Sviri, Raphael Romano Bruno, Bernhard Wernly, Bernardo Bollen Pinto, Joerg C. Schefold, Dorota Studzińska, Michael Joannidis, Sandra Oeyen, Brian Marsh, Finn H. Andersen, Rui Moreno, Maurizio Cecconi, Hans Flaatten, Christian Jung, Wojciech Szczeklik, COVIP & VIP2 study groups

**Affiliations:** 1 Center for Intensive Care and Perioperative Medicine, Jagiellonian University Medical College, ul. Wrocławska 1-3, 30-901 Kraków, Poland; 2 Department of Pulmonology, Jagiellonian University Medical College, Kraków, Poland; 3 UMR_S 1136, Equipe: Epidémiologie Hospitalière Qualité et Organisation des Soins, UPMC Univ Paris 06, INSERMInstitut Pierre Louis d’Epidémiologie et de Santé PubliqueAssistance Publique - Hôpitaux de Paris, Sorbonne Universités, F-75012 Paris, France; 4 Critical Care Department, Corporacion Sanitaria Universitaria Parc Tauli, CIBER Enfermedades Respiratorias, Autonomous University of Barcelona, Sabadell, Spain; 5 Department of Intensive Care Medicine, University Medical Center, University Utrecht, Utrecht, The Netherlands; 6 Department of Anaesthesia and Intensive Care, Viborg Regional Hospital, Viborg, Denmark; 7 Department of Critical Care, St George’s Hospital, London, UK; 8 Medical Intensive Care Unit, Hadassah Medical Center, Jerusalem, Israel; 9 Department of Medical Intensive Care, Hadassah Medical Center and Faculty of Medicine, Hebrew University of Jerusalem, Jerusalem, Israel; 10 Department of Cardiology, Pulmonology and Vascular Medicine, Medical Faculty, Heinrich-Heine-University Duesseldorf, Moorenstraße 5, 40225 Duesseldorf, Germany; 11 Department of Internal Medicine, General Hospital Oberndorf, Teaching Hospital of the Paracelsus Medical University Salzburg, Salzburg, Austria; 12 Institute of General Practice, Family Medicine and Preventive Medicine, Paracelsus Medical University, Salzburg, Austria; 13 Department of Acute Medicine, Geneva University Hospitals, Geneva, Switzerland; 14 Department of Intensive Care Medicine, Inselspital, Bern University Hospital, University of Bern, Bern, Switzerland; 15 Division of Intensive Care and Emergency Medicine, Department of Internal Medicine, Medical University Innsbruck, Innsbruck, Austria; 16 Department of Intensive Care 1K12IC, Ghent University Hospital, Ghent, Belgium; 17 Mater Misericordiae University Hospital, Dublin, Ireland; 18 Department of Anaesthesia and Intensive Care, Ålesund Hospital, Ålesund, Norway; 19 Department of Health Sciences, Norwegian University of Science and Technology, Ålesund, Norway; 20 Faculdade de Ciências Médicas de Lisboa (Nova Médical School), Hospital de São José, Centro Hospitalar Universitário de Lisboa Central, Lisbon, Portugal; 21 Faculdade de Ciências da Saúde, Universidade da Beira Interior, Covilhã, Portugal; 22 Department of Biomedical Sciences, Humanitas University Pieve Emanuele, Milan, Italy; 23 Department of Anaesthesia and Intensive Care, IRCCS Humanitas Research Hospital, Rozzano, Milan Italy; 24 Department of Anaesthesia and Intensive Care, Haukeland University Hospital, Bergen, Norway; 25 Department of Clinical Medicine, University of Bergen, Bergen, Norway

**Keywords:** COVID-19, Older patients, Intensive care unit, Non-invasive ventilation, Respiratory failure

## Abstract

**Background:**

Non-invasive ventilation (NIV) has been commonly used to treat acute respiratory failure due to COVID-19. In this study we aimed to compare outcomes of older critically ill patients treated with NIV before and during the COVID-19 pandemic.

**Methods:**

We analysed a merged cohort of older adults admitted to intensive care units (ICUs) due to respiratory failure. Patients were enrolled into one of two prospective observational studies: before COVID-19 (VIP2—2018 to 2019) and admitted due to COVID-19 (COVIP—March 2020 to January 2023). The outcomes included: 30-day mortality, intubation rate and NIV failure (death or intubation within 30 days).

**Results:**

The final cohort included 1986 patients (1292 from VIP2, 694 from COVIP) with a median age of 83 years. NIV was used as a primary mode of respiratory support in 697 participants (35.1%). ICU admission due to COVID-19 was associated with an increased 30-day mortality (65.5% vs. 36.5%, HR 2.18, 95% CI 1.71 to 2.77), more frequent intubation (36.9% vs. 17.5%, OR 2.63, 95% CI 1.74 to 3.99) and NIV failure (76.2% vs. 45.3%, OR 4.21, 95% CI 2.84 to 6.34) compared to non-COVID causes of respiratory failure. Sensitivity analysis after exclusion of patients in whom life supporting treatment limitation was introduced during primary NIV confirmed higher 30-day mortality in patients with COVID-19 (52.5% vs. 23.4%, HR 2.64, 95% CI 1.83 to 3.80).

**Conclusion:**

The outcomes of patients aged ≥80 years treated with NIV during COVID-19 pandemic were worse compared then those treated with NIV in the pre-pandemic era.

**Supplementary Information:**

The online version contains supplementary material available at 10.1186/s13613-023-01173-2.

## Introduction

Acute respiratory distress syndrome (ARDS) is one of the leading causes of admission to intensive care units (ICUs) around the world [1]. In recent years this trend was strengthened by the COVID-19 pandemic resulting in an overwhelming number of patients admitted to ICUs due to ARDS secondary to SARS-CoV2 infection. The majority of patients suffering from ARDS require invasive mechanical ventilation, but the some experts and guidelines support a trial of non-invasive respiratory support (e.g. NIV) as an appropriate management in selected cases of mild ARDS [2, 3]. The Authors of recently published ARDS management guidelines issued by European Society of Intensive Care Medicine were unable to make a recommendation for or against the use of NIV for the treatment of ARDS due to insufficient evidence[4]. However, due to the shortage of resources in many healthcare systems clinicians have had to resort to using NIV, often outside the ICU, in patients who would typically be intubated in normal circumstances [5].

The frequency of NIV failure in patients with non-COVID ARDS was relatively high and correlated with ARDS severity [6]. Initial reports confirmed NIV to have a low success rate in the treatment of hypoxemic respiratory failure due to COVID-19 [6, 7]. Randomised controlled trials showed, albeit inconsistently, that NIV compared to high-flow oxygen therapy and conventional oxygen therapy reduced the need for intubation but did not reduce mortality [8–10]. Despite limited effectiveness, NIV offers some indisputable advantages compared to invasive mechanical ventilation such as the reduction of invasive procedures associated with patient`s discomfort such as intubation, which is particularly relevant in populations with poor prognosis at baseline [11].

The management of older patients in the ICU is challenging and associated with a high risk of failure largely determined by the degree of frailty at admission [12–14]. Therefore, in many cases, care is centred around patients` comfort and avoidance of invasive procedures whenever possible. To date, there are no available reports describing the use of NIV in very old ICU patients in terms of impact of the COVID-19 pandemic on its frequency, application, and outcomes.

In this analysis of data from two large, prospective, observational studies we aimed to compare outcomes of patients aged ≥80 years primarily treated with NIV before and during the COVID-19 pandemic. Additionally, we describe temporal trends in the frequency and application of NIV in this population.

## Methods

### Study design

The study cohort includes patients enrolled in two prospective observational studies aimed at the evaluation of very old intensive care patients i.e. Prognostic Score in the Very Old ICU Patients (VIP2 study, NCT0337069) and Outcomes and Prognostic Factors in COVID-19 (COVIP study, NCT04321265) [13–15]. Intensive care units participating in the VIP2 study recruited consecutive patients ≥80 years during a 6-month period in 2018–2019. In COVIP patients aged ≥70 years with COVID-19 were recruited from March 2020 to January 2023. Both studies were endorsed by the European Society of Intensive Care Medicine.

The recruitment of ICUs, coordination of national and local ethical permission, and the supervision of patient recruitment at the national level was the responsibility of national coordinators. Ethical approval was mandatory for the study participation in each country. The ethical consent procedures varied across participating countries [16]. In some countries recruitment without informed consent was allowed, while in others the collection of informed consent was mandatory. Patients were followed up to 30 days after ICU admission or until death.

### Study population

For this substudy we excluded patients: (1) aged <80 years enrolled in the COVIP study; (2) patients with reasons for ICU admission other than “respiratory failure” and “combined respiratory and circulatory failure” enrolled in the VIP2 study; (3) lost to 30-day follow-up; (4) with incomplete data on NIV use and (5) recruited in ICUs outside the Europe and Israel.

Each patient was entered only once into the database regardless of readmission, transfer, or other reason. Day 1 of the first admission to an ICU was the reference date. All consecutive dates were numbered sequentially from the admission date.

### Data collection

Data were collected online using electronic case report forms (eCRF). The eCRF and database ran on a secure server composed and stored at Aarhus University, Aarhus, Denmark.

The Sequential Organ Failure Scale (SOFA) score on admission was calculated manually or using an online calculator built into the eCRF [17]. The baseline frailty level was assessed using the Clinical Frailty Scale (CFS) version 1.0. The CFS categorises patients into nine classes from very fit to terminally ill [1–9]. The information on patients` frailty was obtained from the patient or the caregiver/family or hospital records. CFS was further categorised into fit (CFS ≤ 3), vulnerable (CFS = 4) and frail (CFS ≥5) [18, 19].

Type and duration of respiratory support were also documented. This included invasive mechanical ventilation (IMV) and NIV. The available information included: the use of each respiratory support modality, day of initiation and duration of mechanical ventilation. Based on this information, patients were divided into primary NIV group (patients in whom NIV was introduced as the first respiratory support modality) and post-extubation NIV group (patients in whom NIV was the secondary respiratory support modality following invasive mechanical ventilation).

Data about limitation of life supporting treatment LLST (withholding or withdrawing) were documented based on international recommendations and national guidelines. We documented the category and timing of LLST introduction.

### Outcome measurement

The primary endpoint was 30-day mortality. Secondary outcomes were: 30-day intubation rate, length of ICU stay, and NIV failure defined as death or intubation within 30 days since admission to the ICU.

### Statistical analysis

Categorical variables were presented as numbers (percentages) and compared using the χ^2^ test, while continuous variables were presented as medians with interquartile ranges (IQR) and compared using the Mann–Whitney test.

The primary and secondary outcomes were described in a subgroup of patients in whom NIV was used as a primary respiratory support modality. Crude mortality was compared using the log-rank test and visualised using Kaplan–Meier curves. The same approach was used to compare mortality between patients primarily treated with invasive mechanical ventilation and those who were intubated after an initial NIV trial.

Multivariable analysis of factors associated with 30-day mortality was performed using Cox proportional hazard model and the independent variables included: study (VIP vs. COVIP), age, sex, SOFA score and CFS at admission. We also investigated factors associated with intubation rate and NIV failure using logistic regression with the same set of independent variables as listed above. Finally, we evaluated association between 30-day mortality and the number of days between NIV introduction and intubation in a Cox proportional hazard model with adjustment for above mentioned confounders.

We performed a sensitivity analysis for which we excluded patients in whom LLST was introduced while patients were on primary NIV. Such an approach allowed us to evaluate the effectiveness of NIV used as a primary respiratory support in patients without “do not intubate” or “do not resuscitate” orders, contrary to NIV being used as a palliative measure in patients with LLST. In this subgroup we compared 30-day mortality, intubation rate and NIV failure rate in a univariable and multivariable analysis using analogous methods to those described above.

No formal sample size calculation prior to these two purely observational studies was performed. This was a complete case analysis. A two-sided p-value < 0.05 was considered statistically significant. Statistical analyses were performed using the R 4.1.0 software (R Development Core Team, Vienna, Austria).

## Results

### Study sample

**Fig. 1:**
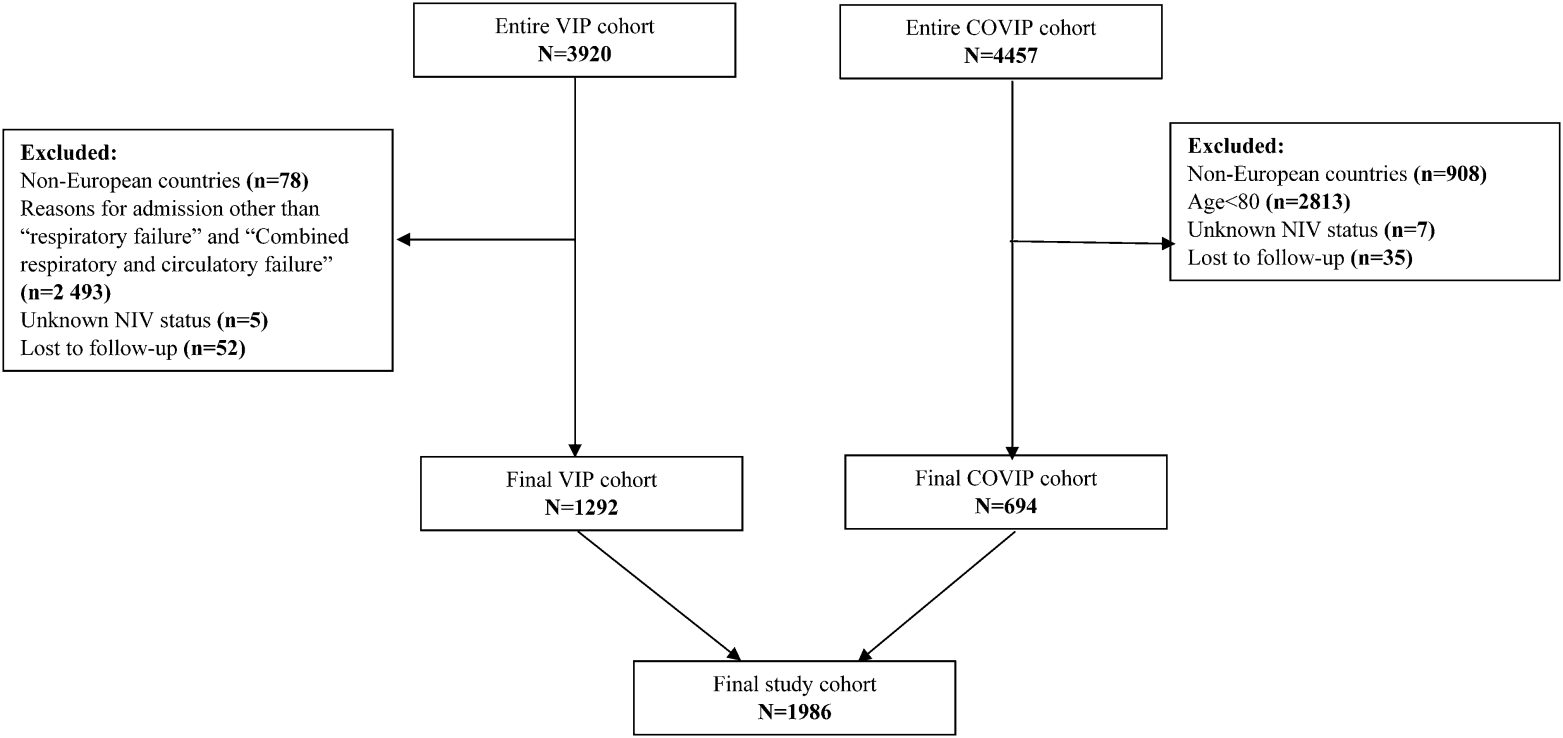
Study flow-chart.

The final cohort of patients consisted of 1986 patients with 1292 patients enrolled in VIP2 study and 694 patients recruited in the COVIP study. Patients included in the COVIP study were younger (83 vs. 84 years, p < 0.001), more frequently male (66.1% vs. 52.8%, p < 0.001) and were less often classified as frail (35.6% vs. 45.2%, p < 0.001) or vulnerable (15.9% vs. 22.7%, p < 0.001). The median follow-up period for the primary outcome was 30 days (IQR 8 to 30). Study flow-chart is presented in Fig. 1.

### Non-invasive ventilation

Non-invasive ventilation was used in 812 patients (40.9%) and less often in the COVIP study (34.3% vs. 44.4%, p < 0.001) with the median day of NIV initiation being the first day of ICU stay for both studies. The duration of NIV therapy was similar in both studies (24.0 vs. 20.0 h, p = 0.121). Among non-invasively ventilated patients, NIV was used as a primary respiratory support modality in 697 participants (86.4%) and as post-extubation respiratory support in 115 patients. The proportion of primary NIV was similar in both studies (86.9% vs. 86.1%, p = 0.856). Comparison of primary NIV patients in COVIP and VIP2 cohorts is presented in Table 1.

**Table 1:** Characteristics of primary NIV patients

Feature	VIP2	COVIP	p-value
**Entire group**			
Number of patients	491	206	
Age	83.00 (81.0 to 86.0)	83.0 (81.0 to 85.0)	0.044
Female sex	251 (51.1)	67 (32.5)	< 0.001
SOFA	4.0 (3.0 to 6.0)	5.0 (3.0 to 7.0)	0.117
CFS	4.0 (3.0 to 6.0)	4.0 (3.0 to 6.0)	0.022
Frailty category			
Fit (CFS ≤ 3)	152 (31.1)	84 (42.9)	
Vulnerable (CFS = 4)	116 (23.7)	29 (14.8)	0.004
Frail (CFS ≥ 5)	221 (45.2)	83 (42.3)	
LLST introduction during NIV	181 (37.3)	83 (40.5)	0.486
30-day mortality	179 (36.5)	135 (65.5)	p < 0.001
Intubation rate	86 (17.5)	76 (36.9)	p < 0.001
NIV failure	222 (45.3)	157 (76.2)	p < 0.001
ICU length of stay	4.0 (1.9 to 7.6)	8.0 (4.0 to 14.8)	p < 0.001
Survivors	4.1 (2.0 to 7.8)	8.5 (5.6 to 21.2)	p < 0.001
Non-survivors	4.0 (1.5 to 7.3)	7.6 (3.1 to 13.3)	p < 0.001
Without LLST on NIV^*^			
Number of patients	304	122	
Age	83.0 (81.0 to 86.0)	83.0 (81.0 to 84.8)	0.087
Female sex	154 (50.7)	41 (33.6)	0.002
SOFA	4.0 (3.0 to 6.0)	5.0 (3.0 to 7.0)	0.022
CFS	4.0 (3.0 to 5.0)	3.0 (3.0 to 5.0)	0.007
Frailty category			
Fit (CFS ≤ 3)	117 (38.6)	63 (54.8)	0.012
Vulnerable (CFS = 4)	74 (24.4)	21 (18.3)	
Frail (CFS ≥ 5)	112 (37.0)	31 (27.0)	
30-day mortality	71 (23.4)	64 (52.5)	< 0.001
Intubation rate	86 (28.3)	76 (62.3)	< 0.001
NIV failure	115 (37.8)	86 (70.5)	< 0.001

Categorical data is presented as n (%), while continuous variables are presented as median (interquartile range)

^*^ Data on LLST was incomplete in 7 patients

In the primary NIV group LLST was introduced during NIV therapy in 264 patients (38.3%) with this proportion being similar in COVIP and VIP2 studies (40.5% vs. 37.3%, p = 0.486). Data on LLST was unavailable in 7 patients.

### Outcomes

**Fig. 2:**
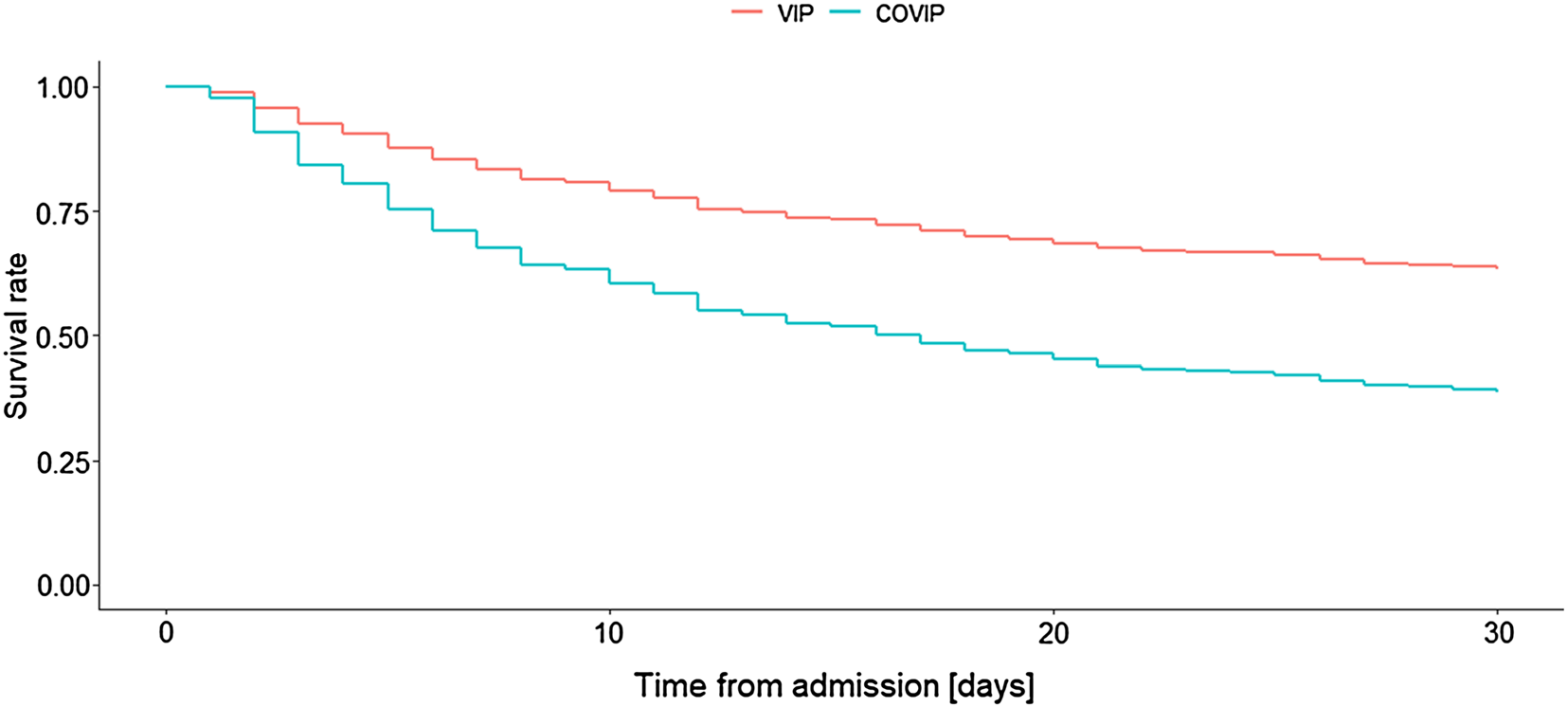
Adjusted survival curves comparing 30-day mortality between patients enrolled in the VIP2 and COVIP studies. Adjustment was made for: age, sex, CFS at admission and SOFA score at admission.

The 30-day mortality rate in the primary NIV group was 45.1% (314/697) and was significantly higher in the COVIP cohort (65.5% vs. 36.5%, log-rank p < 0.001). Multivariable analysis adjusted for age, sex, SOFA and CFS scores confirmed that COVID patients were at higher risk of dying within 30 days of admission compared to patients without SARS-CoV2 infection (HR 2.18, 95% CI 1.71 to 2.77, see Fig. 2).

Among patients in the primary NIV group the intubation rate was 23.2% (162/697) and was significantly higher in patients with confirmed SARS-CoV2 infection (36.9% vs. 17.5%, p < 0.001) compared to the VIP2 cohort. This association was confirmed in the multivariable analysis (OR 2.63, 95% CI 1.74 to 3.99). The number of days between NIV introduction and intubation was higher in the COVIP cohort (1.0 vs. 0.5 days, p = 0.001).

**Fig. 3:**
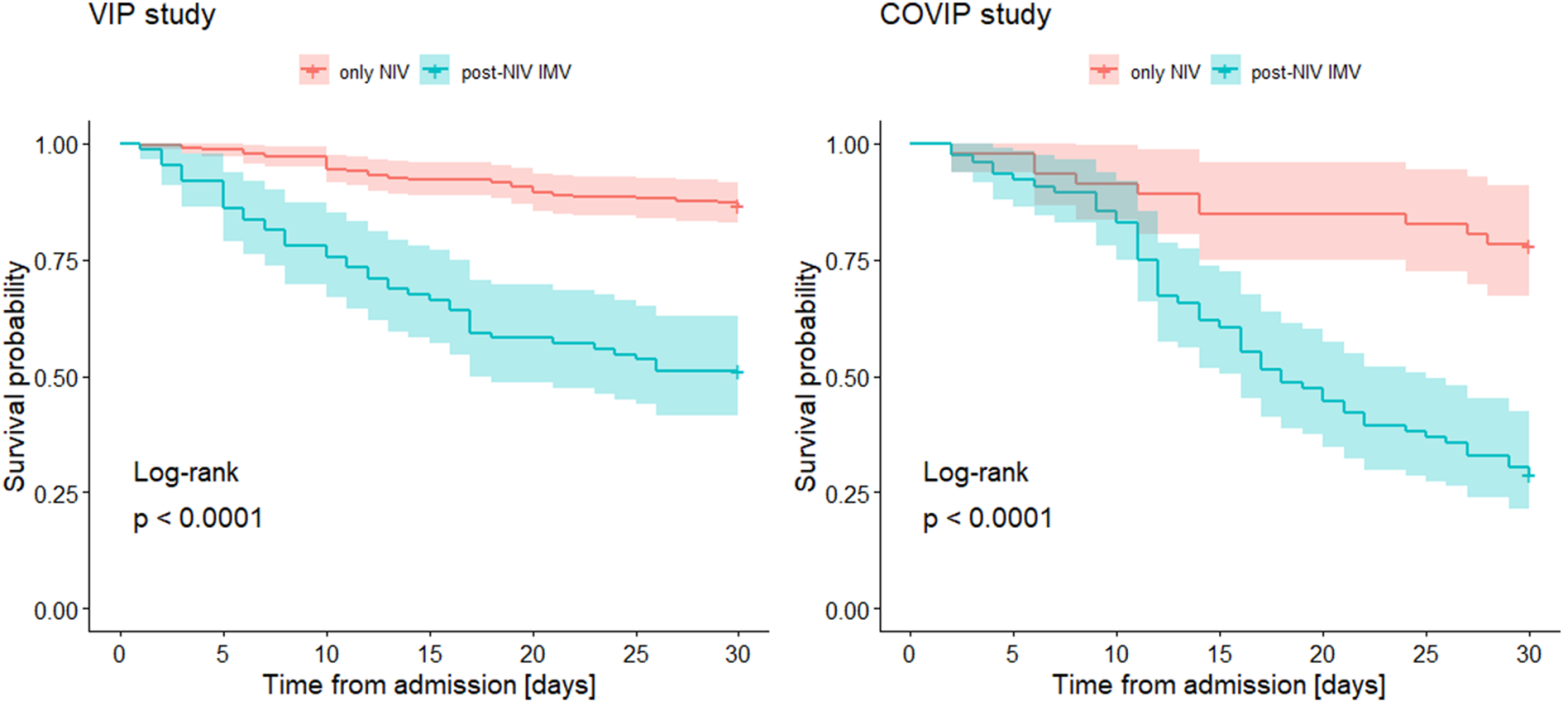
Kaplan–Meier curves comparing a crude 30-day mortality between patients treated initially with NIV who required and who did not require intubation, stratified by study. This curves represent pint estimates with 95% confidence intervals

The mortality rate in patients who eventually required intubation was 59.3% (94/157) and was significantly higher in patients from the COVIP cohort (71.1% vs. 48.8%, log-rank p = 0.027). The requirement for escalation to invasive mechanical ventilation after initial NIV trial was associated with an increased 30-day mortality in both the VIP2 (HR 4.90, 95% CI 2.89 to 8.30) and COVIP (HR 5.60, 95% CI 2.50 to 12.50) cohorts (see Fig. 3) . There was no significant difference in 30-day mortality between patients primarily treated with invasive mechanical ventilation (n = 883) and patients who required intubation after initial NIV trial in the entire cohort (54.5% vs. 59.3%, log-rank p = 0.89) , COVIP cohort (64.8% vs. 71.1%, log-rank p = 0.89) and VIP cohort (49.1% vs. 48.8%, log-rank p = 0.63)—see Additional file 1: Fig. S1. Moreover, after adjustment for age, sex, SOFA and CFS scores, we did not find any association between 30-day mortality and number of days between NIV introduction and intubation for the entire cohort (HR 0.75, 95% CI 0.49 to 1.16), COVIP cohort (HR 0.90, 95% CI 0.49 to 1.63) and VIP cohort (HR 0.91, 95% CI 0.43 to 1.91).

Patients with COVID-19 were hospitalised in the ICU for a longer period of time compared to participants without COVID-19 (8.0 vs. 4.0 days, p < 0.001), both in non-survivors (7.6 vs. 4.0 days, p < 0.001) and survivors (8.5 vs. 4.1 days, p < 0.001).

NIV failure rate was 54.5% (379/697) and was markedly higher in the COVIP study (76.2% vs. 45.3%, p < 0.001). Multivariable analysis confirmed a higher odds of NIV failure in the COVIP cohort (OR 4.21, 95% CI 2.84 to 6.34). Another risk factor for NIV failure was higher SOFA score on admission (OR 1.22, 95% CI 1.14 to 1.31). A summary of outcome analyses is presented in Table 2.

**Table 2:** Summary of outcome analyses

Factor	Outcomes			
	30-day mortality, HR (95% CI)	Intubation rate, OR (95% CI)	NIV failure, OR (95% CI)	30-day mortality (sensitivity analysis), HR (95% CI)
Age	1.04 (1.01 to 1.07)	0.86 (0.80 to 0.92)	1.00 (0.96 to 1.05)	1.02 (0.96 to 1.08)
Female sex	0.81 (0.63 to 1.03)	1.30 (0.86 to 1.99)	0.90 (0.64 to 1.27)	0.84 (0.58 to 1.22)
SOFA	1.08 (1.04 to 1.13)	1.27 (1.18 to 1.37)	1.22 (1.14 to 1.31)	1.10 (1.03 to 1.17)
CFS	1.16 (1.08 to 1.24)	0.78 (0.69 to 0.88)	1.09 (0.98 to 1.20)	1.11 (0.99 to 1.24)
Study (COVIP vs. VIP2)	2.18 (1.71 to 2.77)	2.63 (1.74 to 3.99)	4.21 (2.84 to 6.34)	2.64 (1.83 to 3.80)

### Sensitivity analysis

For sensitivity analysis we compared 30-day mortality in a subgroup of patients in whom LLST was not introduced while on NIV (n = 426). The 30-day mortality rate, intubation rate and NIV failure rates were 31.7% (135/426), 38.0% (162/426) and 47.2% (201/426) respectively. It confirmed a higher mortality rate in the COVIP cohort both in univariable (52.5% vs. 23.4%, p < 0.001) and multivariable analyses (HR 2.64, 95% CI 1.83 to 3.80). Similar results were found in the sensitivity analyses for intubation (62.3% vs. 28.3%, p < 0.001; OR 4.54, 95%CI 2.74 to 7.62) and NIV failure (70.5% vs. 37.8%, p < 0.001; OR 4.05, 95% CI 2.47 to 6.78).

## Discussion

In this analysis of two large observational studies including 1986 patients aged ≥80 years old we showed that, compared to the pre-pandemic era, patients with COVID-19 ARDS treated primarily with NIV were less likely to survive 30 days after admission to the ICU despite being less frail. Other important observations include lower NIV utilisation and more than a quadrupled risk of NIV failure during the COVID-19 pandemic.

Within the first weeks of the pandemic it became clear that COVID-19 was a complex and challenging disease presenting with severe and difficult to treat respiratory failure, increased risk of thrombotic complications and above all high infectivity [20]. This in turn led to nearly immediate exhaustion of global ICU capacity and requirement to maximally optimise distribution of already scarce resources. One of the greatest challenges faced by clinicians and policymakers was managing the constant rapidly escalating requirement for ICU beds. This led to difficult decisions, with some countries resorting to using age as a sole criterion for ICU admission [21]. This was contradictory to accumulating evidence that frailty, rather than age, is a crucial determinant of outcomes in this population, both before and during the pandemic [13, 14, 22]. Very old ICU patients are a particularly fragile and challenging population of patients with limited physiological reserves and poor baseline prognosis. They require careful consideration in terms of the goals of care and the scope of invasive procedures offering potential benefit for these patients. NIV is an excellent example of minimising discomfort in critically ill older patients. It can be used as a respiratory support in both hypoxemic and hypercapnic acute respiratory failure without the need for intubation, neuromuscular blockade and in majority of cases without sedation. For these reasons it was considered a promising alternative to invasive mechanical ventilation during the pandemic crisis.

According to the current guidelines NIV is primarily used in patients with acute COPD exacerbation with hypercapnia and in patients with acute respiratory failure secondary to pulmonary oedema. Due to shortage of ICU beds and personnel as well as relative simplicity of NIV compared to invasive mechanical ventilation, NIV was commonly used outside the ICUs to treat even severe cases of ARDS resulting from COVID-19, which enabled a more optimal distribution of limited ICU resources in this difficult time. Our study confirms that compared to the pre-pandemic era the utilisation of NIV decreased during the pandemic as compared to patients admitted due to respiratory failure in the earlier period [23]. In this analysis we focused more on the application of NIV and found that the proportion of NIV used as a primary respiratory support compared to post-extubation NIV remained the same during the pandemic. We believe that the surprising decrease of NIV utilisation in older ICU patients was mainly driven by the staggering number of patients who required invasive mechanical ventilation which in turn shifted the management of ARDS requiring high-flow nasal oxygen therapy (HFNOT) or NIV outside the ICU. The lower use of NIV in COVID-19 patients was also driven by recommendations for early intubation which appeared in the early phase of the pandemic as well as fear of increased risk of infections affecting medical staff associated with aerosol-generating procedures such as HFNOT or NIV [24]. Unfortunately, we did not gather data about pre-ICU management of respiratory failure and are unable to determine whether and for how long HFNOT or NIV were used in the study participants before the ICU admission.

Regardless of age group the prognosis of patients admitted to the ICU due to COVID-19 is clearly worse compared to other viral or bacterial pneumonias [25, 26]. A recent paper written by the VIP project group confirmed that the survival rate of critically ill patients aged ≥ 80 years nearly halved during the pandemic despite younger age, better functional status and lower severity of organ failure at admission [23]. Our analysis of a subpopulation of patients primarily treated with NIV proves that patients with COVID-19, despite being more frequently categorised as fit according to CFS, had more than a two-time higher risk of dying within 30 days from admission. This observation was confirmed in a sensitivity analysis excluding patients in whom LLST was introduced during NIV. Contrary to the report of the entire population of VIP2 and COVIP studies, analysis of patients primarily treated with NIV revealed a similar rate of LLST in COVID-19 and non-COVID-19 patients. This suggests that the difference observed by Guidet et al. was mostly driven by LLST introduced during invasive ventilation [23]. Worse outcomes of COVID-19 patients treated with NIV are not surprising when one remembers that this technique is not the first choice of therapy in hypoxemic respiratory failure due to pneumonia and in the majority of COVID-19 cases was selected based on logistical, rather than medical reasons. Conversely, VIP2 study population likely included a proportion of patients admitted to the ICU with respiratory failure secondary to acute exacerbation of chronic obstructive pulmonary disease or pulmonary oedema i.e. two indications with the strongest evidence for NIV benefit [27]. To improve comparability of these samples we used multivariable analysis allowing to account for at least part of clinically relevant confounders. Nevertheless, the reader must keep in mind that at least part of the difference in clinical outcomes could be attributed to different indications for NIV therapy.

In the pre-pandemic circumstances intubation rate in patients with ARDS ranged from 22.2% in mild cases to 47.1% in severe cases [6]. The intubation rate in our study was 23.2% and increased to 38.0% after exclusion of patients with LLST. Requirement for intubation after the initial NIV trial has serious clinical implications and is associated with approximately a five times higher risk of death compared to patients who were not intubated. In this subgroup the 30-day mortality reached nearly 50% in non-COVID patients and more than 70% in patients with COVID-19. This may be a least partially related to longer exposure to NIV which is associated with patient self-inflicted lung injury potentially leading to worse outcomes [7, 28]. On the other hand, our analyses showed that the 30-day mortality of patients who required invasive mechanical ventilation was similar regardless of previous NIV attempt in both cohorts while some previous studies showed that early intubation may even be associated with worse outcomes [29]. The optimal timing of intubation remains unclear and requires further research. Due to the specific population of our study we broadened the definition of NIV failure by adding death. We revealed that in approximately half of the cases NIV failed to protect older patients from intubation or death and this proportion was especially high in patients with COVID-19. In this subgroup three out of four patients died or required intubation. A previous report by our group, based on a cohort of COVID-19 patients aged ≥70 years, suggested that increasing duration of NIV prior to intubation was related to higher mortality [7]. The current report does not confirm this observation, but the number of patients included in this analysis was low hence limiting its statistical power. Considering the entire available body of evidence, we believe that when deciding to initiate respiratory support for respiratory failure, especially secondary to SARS-CoV2 infections, clinicians should be aware of the high risk of failure and be ready to intubate and should not delay this decision. Further studies are required to determine the optimal timing and thresholds for escalation from NIV to invasive respiratory support.

The main strength of this paper is it is the prospectively gathered and large sample of homogenous population of critically ill, older patients with a high follow-up completion rate. Precisely gathered data on LLST allowed us to reliably incorporate this crucial aspect of geriatric critical care into our analyses. We are however aware of several limitations of our study. First, we did not gather data on HFNOT which is another form of advanced non-invasive respiratory support which gained a lot of attention during the COVID-19 pandemic. Second, we do not have information about the management of acute respiratory failure prior to ICU admission and therefore we are unable to determine whether NIV or HFNOT were previously used. Third, due to the pragmatic character of this study we were unable to gather some crucial date on NIV such as mode (CPAP vs. bi-level), interface (nasal mask vs. face mask vs. helmet) and complications (e.g. pressure sores). This information would be very valuable from a clinical point of view. Fourth, we had limited data on patients’ characteristic e.g., we did not collect precise data on comorbidities, body mass index, oxygenation index, severity of dyspnoea, and medications that could further reduce the risk of residual confounding. Fifth, lack of precise data about etiology of acute respiratory failure (ARDS vs. COPD exacerbation or pulmonary oedema) prevented us from performing some potentially significant subgroup analyses. Sixth, we did not collect data on post-ICU care trajectory and hence were unable to determine the LLST status after transfer from the ICU and the reasons of death in ICU survivors who died within 30 days. Finally, this study includes only older critically ill patients with COVID-19 and therefore the generalizability of the results outside this group is limited.

Presented analysis of merged data from VIP2 and COVIP studies revealed that older critically ill patients with COVID-19 initially treated with NIV are at higher risk of dying within 30 days of ICU admission compared with patients treated for respiratory failure of different aetiologies. We also confirmed that the odds of NIV success were low in patients aged ≥80 and decreased dramatically during the pandemic. The causes of worse outcomes in COVID-19 patients are likely to be multifactorial and at least partially related to the scarcity of resources faced in the early phase of the pandemic.

### Supplementary Information

**Additional file 1:** **Fig. S1.** Kaplan–Meier curves for comparison of mortality in patients undergoing primary invasive mechanical ventilation and post-NIV invasive mechanical ventilation. **Table S1.** Countries recruiting patients in VIP2 and COVIP studies.

## Data Availability

The datasets used and/or analysed during the current study are available from the corresponding author on reasonable request.

## Abbreviations

NIV: Non-invasive ventilation
COVID-19: Coronavirus disease 2019
ICUS: Intensive care units
ARDS: Acute respiratory distress syndrome
VIP2 study: Prognostic score in the very old ICU patients
COVIP: Outcomes and prognostic factors in COVID-19
eCRF: Electronic case report forms
SOFA: The sequential organ failure scale
CFS: The clinical frailty scale
IMV: Invasive mechanical ventilation
LLST: Limitation of life supporting treatment
IQR: Interquartile ranges
HR: Hazard ratio
OR: Odds ratio
CI: Confidence interval
HFNOT: High-flow nasal oxygen therapy
CPAP: Continuous positive airway pressure
