# Supplementary material for: Outcomes of patients aged ≥80 years with respiratory failure initially treated with non-invasive ventilation in European intensive care units before and during COVID-19 pandemic
Source: Ann Intensive Care. 2023 Sep 12;13:82. doi: 10.1186/s13613-023-01173-2 (PMC10497468; doi:10.1186/s13613-023-01173-2)

**ADDITIONAL MATERIAL**

**List of contents:**

**Additional Table 1.** Countries recruiting patients in VIP2 and COVIP studies.

**Additional Figure 1.** Kaplan-Meier curves for comparison of mortality in patients undergoing primary invasive mechanical ventilation and post-NIV invasive mechanical ventilation.

**Additional Table 1.** Countries recruiting patients in VIP2 and COVIP studies.

| **Country** | **COVIP** | | **VIP** | |
| --- | --- | --- | --- | --- |
|  | **Cohort** | **Primary NIV** | **Cohort** | **Primary NIV** |
| Austria | 7 | 2 | 12 | 8 |
| Belgium | 43 | 1 | 24 | 8 |
| Switzerland | 66 | 22 | 20 | 12 |
| Germany | 146 | 65 | 45 | 24 |
| Denmark | 55 | 21 | 77 | 37 |
| England | 41 | 20 | 231 | 95 |
| Spain | 25 | 0 | 63 | 24 |
| France | 148 | 37 | 303 | 157 |
| Greece | 54 | 10 | 107 | 10 |
| Ireland | 1 | 0 | 16 | 6 |
| Israel | 22 | 7 | 0 | 0 |
| Italy | 1 | 1 | 25 | 8 |
| The Netherlands | 21 | 0 | 56 | 12 |
| Norway | 3 | 3 | 70 | 49 |
| Poland | 31 | 2 | 144 | 6 |
| Portugal | 25 | 15 | 50 | 12 |
| Wales | 5 | 0 | 4 | 1 |
| Croatia | 0 | 0 | 1 | 0 |
| Sweden | 0 | 0 | 42 | 22 |
| Ukraine | 0 | 0 | 2 | 0 |

**Additional Figure 1.** Kaplan-Meier curves for comparison of mortality in patients undergoing primary invasive mechanical ventilation and post-NIV invasive mechanical ventilation.


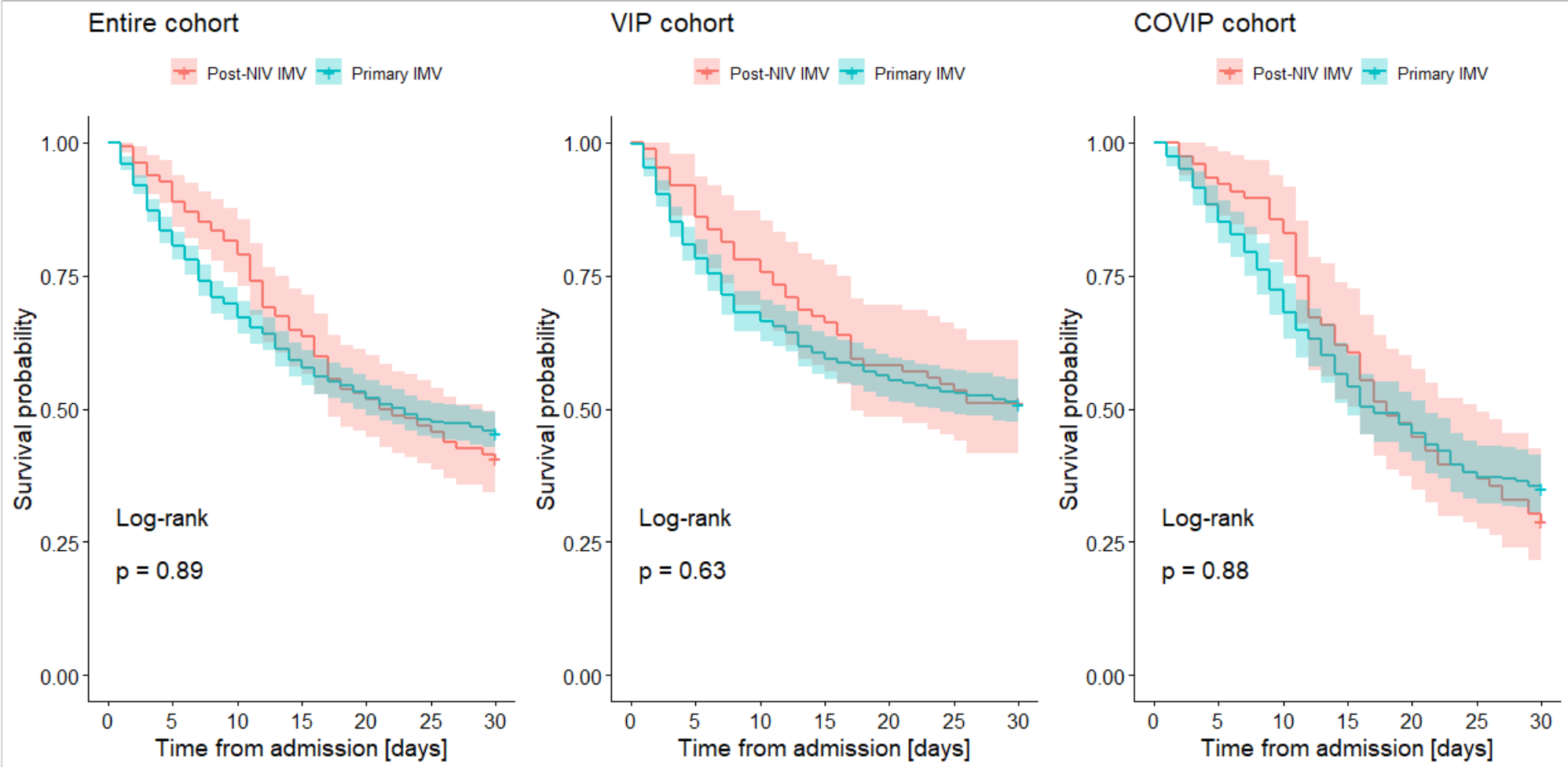

Supplement: Supplementary file 1 — Additional file 1: Fig. S1. Kaplan–Meier curves for comparison of mortality in patients undergoing primary invasive mechanical ventilation and post-NIV invasive mechanical ventilation. Table S1. Countries recruiting patients in VIP2 and COVIP studies. [file 13613_2023_1173_MOESM1_ESM.docx]
